# Comparing Precision Machine Learning With Consumer, Quality, and Volume Metrics for Ranking Orthopedic Surgery Hospitals: Retrospective Study

**DOI:** 10.2196/22765

**Published:** 2020-12-01

**Authors:** Dev Goyal, John Guttag, Zeeshan Syed, Rudra Mehta, Zahoor Elahi, Mohammed Saeed

**Affiliations:** 1 Health at Scale Corporation San Jose, CA United States; 2 Department of Electrical Engineering and Computer Science Massachusetts Institute of Technology Cambridge, MA United States; 3 Department of Internal Medicine University of Michigan Ann Arbor, MI United States

**Keywords:** machine learning, hospital ratings, precision delivery, hospital, surgery, outcome, perioperative, internet, reputation, machine learning

## Abstract

**Background:**

Patients’ choices of providers when undergoing elective surgeries significantly impact both perioperative outcomes and costs. There exist a variety of approaches that are available to patients for evaluating between different hospital choices.

**Objective:**

This paper aims to compare differences in outcomes and costs between hospitals ranked using popular internet-based consumer ratings, quality stars, reputation rankings, average volumes, average outcomes, and precision machine learning–based rankings for hospital settings performing hip replacements in a large metropolitan area.

**Methods:**

Retrospective data from 4192 hip replacement surgeries among Medicare beneficiaries in 2018 in a the Chicago metropolitan area were analyzed for variations in outcomes (90-day postprocedure hospitalizations and emergency department visits) and costs (90-day total cost of care) between hospitals ranked through multiple approaches: internet-based consumer ratings, quality stars, reputation rankings, average yearly surgical volume, average outcome rates, and machine learning–based rankings. The average rates of outcomes and costs were compared between the patients who underwent surgery at a hospital using each ranking approach in unadjusted and propensity-based adjusted comparisons.

**Results:**

Only a minority of patients (1159/4192, 27.6% to 2078/4192, 49.6%) were found to be matched to higher-ranked hospitals for each of the different approaches. Of the approaches considered, hip replacements at hospitals that were more highly ranked by consumer ratings, quality stars, and machine learning were all consistently associated with improvements in outcomes and costs in both adjusted and unadjusted analyses. The improvement was greatest across all metrics and analyses for machine learning–based rankings.

**Conclusions:**

There may be a substantive opportunity to increase the number of patients matched to appropriate hospitals across a broad variety of ranking approaches. Elective hip replacement surgeries performed at hospitals where patients were matched based on patient-specific machine learning were associated with better outcomes and lower total costs of care.

## Introduction

Patients undergoing elective surgeries often seek information at different levels of granularity when choosing providers, ranging from institutions to practices to individual physicians. It is widely established that the choice of provider significantly impacts both perioperative outcomes and costs [1-4]. There exist a variety of approaches that are available to patients for evaluating between different provider choices. These include consumer ratings, government quality ratings (eg, Centers for Medicare & Medicaid Services [CMS] stars), reputation rankings, and average volumes and outcomes [5-17]. Machine learning–based online tools are emerging as an alternative method of predicting provider performance that can factor patient-specific characteristics into provider rankings [17,18]. Unfortunately, there is little prior research into the comparative performance of these methods in predicting outcomes associated with different providers [19-21].

This study compares different approaches (consumer ratings, CMS quality stars, reputation rankings, and average volumes and outcomes) and personalized patient-specific rankings using machine learning (referred to as precision navigation) for predicting hospital performance, as measured by postprocedure hospitalization rate, emergency department (ED) visits, and total cost of care. The analyses were performed on data from Medicare fee-for-service (FFS) beneficiaries following elective hip replacement surgery. For each of the different approaches, 90-day outcomes for patients that were treated at highly ranked institutions were measured and compared to the outcomes for patients treated at institutions that were not highly ranked. Outcomes following elective hip replacement surgeries were studied because they are among the most commonly performed surgeries and because there is considerable variation in risk-adjusted performance across hospitals. Postprocedure hospitalization, ED visits, and total cost of care were used as outcome measures because they are quantifiable, meaningful to patients, and available for Medicare FFS populations.

## Methods

### Study Population

The study population comprised Medicare FFS beneficiaries undergoing elective hip replacements between 2013 to 2018 in the Chicago-Naperville-Arlington Heights, Illinois metropolitan area. Hip replacement surgeries were identified among these patients using standard procedure codes available in administrative claims data.

All hospitals in the greater Chicago metropolitan area that were visited for hip replacement surgery by at least one Medicare FFS beneficiary who lived in the Chicago-Naperville-Arlington Heights, Illinois area between 2016 and 2018 were included in the analysis (thus, for all hospitals that were active between 2016 and 2018, all data from between 2013 and 2018 were used in the analysis). For each patient, up to 10 of the nearest candidate hospitals that were a maximum of 50 miles from their place of surgery were ranked. For each patient, the top-ranked choice was determined for the different approaches, and the average outcomes and costs for patients who had surgeries at a top-ranked hospital were compared with the population averages.

### Ranking Methodologies

#### Ratings

For each hospital, online consumer ratings from Yelp [6], Healthgrades [22], and US News [19] and quality star ratings from CMS Hospital Compare [10] were used for ranking purposes. The 2019 ratings available at the time of conducting this study were used for each approach in the absence of historical values for these approaches. Yelp and CMS Hospital Compare included overall hospital ratings scored between 1 and 5 (rather than specialty-specific ratings). Healthgrades online reports included ratings for hospital performance specific to hip replacement procedures. US News ratings for orthopedic surgery for each hospital were used. If two or more hospitals had the same ratings, the hospital with the larger number of reviews (or patient volume in the case of CMS ratings) was considered the top-ranked hospital.

#### Statistics

Procedural volumes were calculated for hip replacement using Medicare FFS data for each hospital between 2013 and 2017. The average postprocedure hospitalization rate and ED admission rate at each hospital following hip replacement procedures were calculated using Medicare FFS data as a measure of quality and were also treated as a rating. For each of these ratings (procedural volume and average rates), a single average was computed from 2013 to 2017. For evaluating volume-based ratings, the top hospital was the one with the highest average yearly volume of hip replacement surgeries. For evaluating average outcome–based ratings, the top hospital was the one with the lowest combined average 90-day postprocedure hospitalization rate and ED admission rate for hip replacement surgery.

#### Precision Navigation

Machine learning–based rankings of orthopedic facilities were generated for each beneficiary undergoing hip replacement in 2018 using a commercially available software system (Precision Navigation; Health at Scale Corp) that uses precision navigation and was trained on data prior to 2018 (ie, from 2013-2017) [18]. The top hospital for each beneficiary was determined as the top-ranked result returned by this system based on the patient’s individual characteristics. The system predicts the hospitals likely to have the best long-term outcomes for patients based on the patient’s personalized medical characteristics by learning from historical data about hospitals’ long-term outcomes on similar patients.

### Outcomes

The 90-day postprocedure hospitalization rate and rate of ED admission following the surgery were measured as outcomes. In addition, the 90-day total cost of care (TCOC) was also measured for each patient, which included the costs of the surgery and subsequent costs for the patient that occurred over the next 90 days from the day of surgery. The TCOC estimate included reimbursements for inpatient, outpatient, skilled nursing facility, and home health agency care, as these were available for the FFS beneficiaries. All patients included in the analyses had at least 90 days of follow-up (ie, follow-up data for patients were available until April 2019).

The observed average outcomes (postprocedure hospitalization rate, ED admission) and TCOC were compared between patients who received treatment at the top-ranked hospital for hip replacement surgery in 2018 and the overall population (all patients who had hip replacement surgery in 2018) in an unadjusted analysis. The unadjusted comparisons were also done in subgroups stratified by the Elixhauser comorbidity score. In each comparison, we statistically compared the outcomes of interest between patients who were admitted to a top-ranked hospital and patients who were admitted to a lower-ranked hospital (as determined by each respective method).

Pairwise comparisons between patients who visited a top-ranked hospital based on the different approaches were made using an adjusted propensity-based analysis with a one-to-one matching caliper without replacement [23]. Propensity was calculated using the weighted Elixhauser comorbidity score (each Elixhauser comorbidity was weighted to calculate a composite score for hospital readmissions) [24]. The propensity-matched groups for each pairwise comparison were generated 100 times by sampling with replacement. Thus, within each pairwise comparison between the various rating approaches, only patients with similar comorbidity scores (based on the matching caliper) were compared.

## Results

Table 1 presents the characteristics of the Medicare FFS beneficiaries in the Chicago metropolitan area that received an inpatient hip replacement in 2018. The data consisted of 4192 hip replacement surgeries performed at 69 total hospitals.

Table 2 presents descriptive summaries for consumer ratings, quality stars, reputation rankings, average yearly surgical volume, average outcome rates, and precision navigation–based ranking for hospitals in the Chicago metropolitan area. The majority of the approaches had ratings for all of the hospitals in the region (all approaches excluding US News and Healthgrades, which covered more than half but not all of the hospitals in the Chicago metropolitan area).

Table 3 compares the outcomes and TCOC of patients who had surgeries at top hospitals with those of the overall population for each approach. Patients who underwent surgeries at hospitals that were ranked as top hospitals according to the CMS quality stars, Yelp, the average volume, the average outcome rate, and the precision navigation–based approaches were associated with substantially better outcomes than the population averages. This improvement in outcomes was greatest for precision navigation. The US News and Healthgrades approaches were associated with improvements in ED admission rates and postprocedure hospital rates or in TCOC but not in both. The precision navigation–based rankings were associated with an improvement of 3.4% for postprocedure hospitalization rate (*P*<.001), 4.1% for ED admission rates (*P*<.001), and US $3315 for TCOC (*P*<.001) between top hospitals and the overall population. The other ranking methods were associated with smaller variations in outcomes between top hospitals and the overall population. The percentage of patients who underwent surgery at a hospital ranked as a top hospital by each ratings approach varied from 27.6% (1159/4192) for the precision navigation–based rankings to 49.6% (2078/4192) for the average volume–based rankings.

**Table 1:** Patient characteristics used in the hip replacement analyses.^a^

Patient characteristics		Value
Patients, n		4192
Age (years), mean (SD)		69.4 (8.2)
Male, n (%)		1589 (38)
**Race/ethnicity, n (%)**		
	White	3602 (86)
	African American	417 (10)
	Unknown	85 (2)
	Other	39 (1)
	Hispanic	28 (1)
	Asian	21 (1)
**Comorbidities, n (%)^b^**		
	Hypertension	1699 (43)
	Anemic deficiency	428 (10)
	Diabetes	653 (16)
	Hypothyroidism	398 (9)
	Chronic lung disease	374 (9)
	Obesity	320 (8)
	Electrolyte imbalance	284 (7)
	Depression	273 (7)
	Arthritis	235 (6)
	Tumors without metastases	223 (5)
**90-day outcomes^c^**		
	ED^d^ admission rate, n (%)	655 (15.6)
	Postprocedure hospitalization rate, n (%)	469 (11.2)
	Reimbursement (US $), mean (SD)	27,000 (21,000)

^a^The study cohort includes all Medicare fee-for-service beneficiaries in the Chicago metropolitan area that underwent an elective hip replacement surgery in 2018.

^b^The 10 most common comorbidities are shown.

^c^ED and postprocedure hospitalizations are calculated as occurring at least once within the 90-day follow-up period.

^d^ED: emergency department.

**Table 2:** A summary of the ranking methods used in this study.

Rating system and characteristic			Value
**US News**			
	Hospitals, n	60	
	Range	0-100	
	Mean (SD)	42 (10)	
**Yelp**			
	Hospitals, n	87	
	Range	1-5	
	Number of reviews, mean	57	
	Mean rating (SD)	2.5 (0.7)	
**Healthgrades**			
	Hospitals, n	75	
	Range	1-5	
	Mean rating (SD)	2.5 (1.2)	
**CMS^a^**			
	Hospitals, n	90	
	Range	1-5	
	Mean (SD)	3.1 (1.3)	
**Average volumes**			
	Hospitals, n	94	
	Range	0-752	
	Mean rating (SD)	153 (156)	
**Average outcomes: ED^b^ admission rate**			
	Hospitals, n	94	
	Range	5-100	
	Mean rating (SD)	18.2 (11.0)	
**Average outcomes: postprocedure hospitalization rate**			
	Hospitals, n	94	
	Range	0-50	
	Mean hip rating (SD)	14.7 (8.1)	
**Precision navigation**			
	Hospitals, n	90	
	Range	1-10	

^a^CMS: Centers for Medicare & Medicaid Services.

^b^ED: emergency department.

**Table 3:** Comparison of average outcomes between patients who went to top-ranked hospitals for hip replacement surgery and the overall population.

Outcome		Precision navigation	US News	Healthgrades	Yelp	CMS^a^	Average volume	Average outcome rate
**90-day ED^b^ admission rate**								
	In-top average^c^, n (%)	486.3 (11.6)	639.8 (15.3)	659.0 (15.7)	585.3 (14.0)	584.4 (13.9)	605.2 (14.4)	584.6 (13.9)
	Population average^d^ – in-top average, n (%)	171.9 (4.1)	15.2 (0.4)	–4.0 (0.1)	69.7 (1.7)	70.6 (1.7)	49.8 (1.2)	70.4 (1.7)
	*P* value	<.001	.55	.91	.03	.009	.04	.03
**90-day postprocedure hospitalization rate**								
	In-top average, n (%)	327.0 (7.8)	413.0 (9.9)	472.6 (11.3)	407.4 (9.7)	396.7 (9.5)	417.6 (10.0)	415.6 (9.9)
	Population average – in-top average, n (%)	142.5 (3.4)	56.0 (1.3)	–3.6 (0.1)	61.6 (1.5)	72.3 (1.7)	51.4 (1.2)	53.4 (1.3)
	*P* value	<.001	.01	.91	.03	.002	.01	.05
**90-day total cost of care**								
	In-top average (US $)	23,698	28,328	26,944	28,088	26,412	28,047	28,067
	Population average – in-top average (US $)	3315	–1315	68	1076	601	1035	1054
	*P* value	<.001	<.001	.005	<.001	.003	<.001	<.001
Patients who visited top hospitals, n (%)		1159 (27.6)	1959 (46.7)	1304 (31.1)	1461 (34.9)	1786 (42.6)	2078 (49.6)	1513 (36.1)

^a^CMS: Centers for Medicare & Medicaid Services.

^b^ED: emergency department.

^c^In-top average: outcome rate among patients who were admitted to top-ranked hospitals using the particular ranking approach.

^d^Population average: average value of the particular outcome in the overall population.

Table 4 presents the average outcomes and TCOC for patients who had surgeries at top hospitals, stratified by their Elixhauser comorbidity scores. For both low (≤0) and high (>0) comorbidity score groups, patients who visited top hospitals using precision navigation–based ranking had the best outcomes and TCOC.

Table 5 compares the outcomes and TCOC of patients in a pairwise, adjusted, propensity-matched analysis. For each pairwise comparison, patients matched to top hospitals using both ranking approaches were propensity matched, and the differences in average outcome rates between these propensity-matched patients were computed. In the pairwise propensity-matched comparison using the precision navigation–based approach with each of the other approaches, the improvement in outcomes was greatest for precision navigation for every outcome and every pairwise comparison.

**Table 4:** Comparison of outcomes between patients who went to top-ranked hospitals for hip replacement surgery and the general population, using different ranking methodologies.

Elixhauser comorbidity score quantile and outcome				Precision navigation		US News		Healthgrades		Yelp		CMS^a^		Average volume		Average outcome rate
≤**0 (low)**																
	Patients who visited top hospitals, n (%)		933.7 (29.4)		1461.0 (46.0)		990.9 (31.2)		1133.8 (35.7)		1349.8 (42.5)		1559.4 (49.1)		1140.2 (35.9)	
	**90-day ED^b^admission rate**															
		In-top average^c^, n (%)	327.1 (10.3)		390.6 (12.3)		463.7 (14.6)		371.6 (11.7)		374.8 (11.8)		368.4 (11.6)		362.1 (11.4)	
		In-bin average – in-top average, n (%)	98.5 (3.1)		31.8 (1.0)		–41.3 (1.3)		54.0 (1.7)		47.6 (1.5)		54.0 (1.7)		60.3 (1.9)	
		*P* value	<.001		.13		.16		.04		.03		.005		.02	
	**90-day postprocedure hospitalization rate**															
		In-top average, n (%)	187.4 (5.9)		219.1 (6.9)		289.0 (9.1)		222.3 (7.0)		225.5 (7.1)		209.6 (6.6)		241.4 (7.6)	
		In-bin average – in-top average, n (%)	88.9 (2.8)		54.0 (1.7)		–12.7 (0.4)		54.0 (1.7)		50.8 (1.6)		63.5 (2.0)		34.9 (1.1)	
		*P* value	<.001		.002		.59		.01		.007		<.001		.10	
	**90-day TCOC^d^**															
		In-top average (US $)	22,963		26,696		26,237		27,045		25,032		26,404		26,713	
		In-bin average – in-top average (US $)	2665		–1068		–610		–1418		595		–776		–1086	
		*P* value	<.001		<.001		.13		<.001		.001		<.001		<.001	
**>0 (high)**																
	Patients who visited top hospitals, n (%)		224.5 (22.1)		498.9 (49.1)		311.9 (30.7)		328.2 (32.3)		434.8 (42.8)		520.2 (51.2)		373.9 (36.8)	
	**90-day ED admission rate**															
		In-top average, n (%)	536.7 (16.9)		755.9 (23.8)		609.8 (19.2)		698.7 (22.0)		651.1 (20.5)		727.3 (22.9)		689.2 (21.7)	
		In-bin average – in-top average, n (%)	184.2 (5.8)		–34.9 (1.1)		111.2 (3.5)		25.4 (0.8)		73.0 (2.3)		–3.2 (0.1)		34.9 (1.1)	
		*P* value	.02		.41		.09		.75		.15		.94		.59	
	**90-day postprocedure hospitalization rate**															
		In-top average, n (%)	495.5 (15.6)		584.4 (18.4)		581.2 (18.3)		609.8 (19.2)		533.6 (16.8)		635.2 (20.0)		543.1 (17.1)	
		In-bin average – in-top average, n (%)	111.2 (3.5)		22.2 (0.7)		25.4 (0.8)		–3.2 (0.1)		73.0 (2.3)		–28.6 (0.9)		63.5 (2.0)	
		*P* value	.15		.63		.73		.99		.11		.47		.25	
	**90-day TCOC**															
		In-top average (US $)	26,748		33,102		29,192		31,691		30,696		32,969		32,189	
		In-bin average – In-top average (US $)	4593		–1761		2149		–350		645		–1628		–848	
		*P* value	<.001		<.001		.003		.63		.64		.003		<.001	

^a^CMS: Centers for Medicare & Medicaid Services.

^b^ED: emergency department.

^c^In-top average: outcome rate among patients who were admitted to top-ranked hospitals using the particular approach.

^d^TCOC: total cost of care.

**Table 5:** A pairwise propensity-matched comparison of patients matched to top hospitals using each of the ranking approaches.

Reduction in outcome rate^a^		Relative to US News		Relative to Healthgrades		Relative to Yelp		Relative to CMS^b^		Relative to average volume		Relative to average outcome rate	
		Outcome	*P* value	Outcome	*P* value	Outcome	*P* value	Outcome	*P* value	Outcome	*P* value	Outcome	*P* value
**Precision navigation**													
	90-day ED^c^ admission rate, %	2.5	<.001	3.8	<.001	2.0	<.001	1.8	<.001	2.1	<.001	1.7	<.001
	90-day postprocedure hospitalization rate, %	1.1	<.001	2.9	<.001	1.4	<.001	1.2	<.001	1.1	<.001	1.6	<.001
	90-day TCOC^d^ (US $)	4025	<.001	2923	<.001	4298	<.001	2309	<.001	3840	<.001	3817	<.001
**US News**													
	90-day ED admission rate, %	N/A^e^	N/A	0.4	<.001	–0.9	<.001	–1.3	<.001	–0.7	<.001	–1.1	<.001
	90-day postprocedure hospitalization rate, %	N/A	N/A	1.5	<.001	–0.1	.87	–0.3	.89	0.3	.43	0.0	.22
	90-day TCOC (US $)	N/A	N/A	–1161	<.001	–269	.32	–1918	<.001	–191	<.001	–299	.001
**Healthgrades**													
	90-day ED admission rate, %	0.4	<.001	N/A	N/A	–1.9	<.001	–1.7	<.001	–0.9	<.001	–1.7	<.001
	90-day postprocedure hospitalization, %	1.5	<.001	N/A	N/A	–1.5	<.001	–1.8	<.001	–1.2	<.001	–1.5	<.001
	90-day TCOC (US $)	–1161	<.001	N/A	N/A	757	<.001	–542	<.001	867	<.001	910	<.001
**Yelp**													
	90-day ED admission rate, %	–0.9	<.001	–1.9	<.001	N/A	N/A	–0.2	.39	0.4	.002	–0.3	.02
	90-day postprocedure hospitalization rate, %	–0.1	.87	–1.5	<.001	N/A	N/A	–0.5	<.001	0.1	.33	0.0	.30
	90-day TCOC (US $)	–269	.32	757	<.001	N/A	N/A	–1693	<.001	–105	.19	–216	.16
**CMS**													
	90-day ED admission rate, %	–1.3	<.001	–1.7	<.001	–0.2	.39	N/A	N/A	0.2	<.001	–0.1	.91
	90-day postprocedure hospitalization rate, %	–0.3	.89	–1.8	<.001	–0.5	<.001	N/A	N/A	0.4	<.001	0.3	.004
	90-day TCOC (US $)	–1918	<.001	–542	<.001	–1693	<.001	N/A	N/A	1486	<.001	1575	<.001
**Average volume**													
	90-day ED admission rate, %	–0.7	<.001	–0.9	<.001	0.4	.002	0.2	<.001	N/A	N/A	–0.5	<.001
	90-day postprocedure hospitalization rate, %	0.3	.43	–1.2	<.001	0.1	.33	0.4	<.001	N/A	N/A	–0.1	.05
	90-day TCOC (US $)	–191	<.001	867	<.001	–105	.19	1486	<.001	N/A	N/A	–104	.86

^a^Differences were calculated as column – row; thus, positive values imply a lower adverse outcome rate.

^b^CMS: Centers for Medicare & Medicaid Services.

^c^ED: emergency department.

^d^TCOC: total cost of care.

^e^N/A: not applicable.

The potential impact of using different rating systems under the counterfactual assumption that patients only presented to a top hospital for them was also considered. The precision navigation–based ranking approach resulted in significantly more hospitals being designated as a top hospital for one or more Medicare FFS beneficiary (ie, distributed or balanced the case load across a larger number of hospitals that were top ranked for specific patients). Out of 69 total hospitals visited by patients in 2018 for hip replacements, 54 were considered top-ranked hospitals for at least one patient when using precision navigation. CMS ratings resulted in 25 hospitals being top ranked. US News and Healthgrades each resulted in 27 hospitals being top ranked. Yelp-based ranking resulted in 22 hospitals being considered top ranked. Average volume and outcome resulted in 26 and 24 hospitals, respectively, being considered top-ranked hospitals for a patient.

## Discussion

Prior research on the use of popular ranking and rating approaches, including web-based ratings, consumer guides, and various quality ratings for physicians or hospitals, have resulted in inconsistent findings, and it is unclear which rating approach works best [5,20,21,25-28]. This retrospective study compares the performance of several different rating strategies for designating top hospitals for a large population of Medicare FFS beneficiaries who underwent elective hip replacement surgeries in the Chicago metropolitan area. The study also compared the performance of the aforementioned approaches for hospital rankings and ratings with a more personalized precision navigation–based approach that selects hospitals based on patients’ individual health characteristics.

Overall, several approaches were shown to be associated with better outcomes and lower TCOC when patients presented to a top hospital based on the respective ranking approach. These included CMS quality stars and precision navigation–based rankings, with top-ranked hospitals achieving improved outcomes and TCOC for hip replacement using both propensity-adjusted and nonadjusted analyses. The greatest improvements were observed for precision navigation–based rankings, which were more consistently associated with reductions in 90-day postprocedure hospitalization rate, ED admission rate, and TCOC in each analysis.

Prior research has resulted in an inconsistent correlation between outcomes and ratings in top-ranked hospitals. For example, Cram et al [29] showed no significant differences in total knee arthroplasty outcomes in top-ranked and non–top-ranked hospitals using the US News rankings. Studies in other surgical subspecialties, such as cardiac surgery, found only a weak correlation between online ratings and perioperative mortality [25]. Osborn et al [27] showed a significant correlation between favorable rankings and lower mortality rates associated with various major surgical procedures. However, use of mortality as a surrogate for outcome may not reflect other pertinent outcomes that occur with higher frequency, such as postprocedure hospital admissions or ED visits.

In this study, hospital performance was measured using the rates of postprocedure hospitalizations and ED visits and the total cost of care among Medicare FFS beneficiaries undergoing elective hip replacements. These metrics, which were available for all hospitals included in this study, are meaningful to patients. Many of the popular consumer-based ranking approaches are based more on patient satisfaction than on objective measures [6,25,26,30]. Velasco et al [31] found that negative online comments about orthopedic surgeons were associated with surgery-independent factors, such as waiting time and logistics. Austin et al [32] showed that there was considerable variation in the rankings of top hospitals when different criteria were used. The significant variation in ratings across the different rating platforms may complicate the choice of institution for patients [7,8,33]. Moreover, all of these approaches implicitly assume that the choice of a top hospital is independent of the characteristics of the patient [19,32]. The results of this study call this assumption into question.

Typical methods for ranking hospitals for specialty procedures assume that the quality of an institution is the same for all patients. The personalized approach of precision navigation–based ranking predicts outcomes for each hospital-patient pair by learning from the respective hospital’s prior outcomes for patients who resemble the patient being matched. Thus, this study suggests that hospital quality may be a personalized, variable phenomenon rather than a global, uniform value. The overwhelming majority of hospitals considered are not consistently ideal (ie, the top choice) for all patients but instead lie largely in a “gray zone” of being selectively good or bad for individual patients. More than 85% (80/94) of the hospitals that were included in the Chicago metropolitan area fell in this category. This finding is perhaps not surprising, given the complexity of surgical patients and the variations in case mixes, resources, training, and other organizational characteristics of hospitals that lead to facilities performing well or poorly for specific individuals. Prior research has shown that patient complexity is strongly correlated with outcomes in elective surgeries, such as total knee arthroplasty [34]. Bozic et al [35] also found considerable variation in respective patient population acuities of hospitals performing elective hip and knee replacements and a fourfold difference in risk-adjusted complication rates [35].

The potential policy implications of our study are significant. The paradigm of patients choosing institutions based on crowdsourced online ratings, popular consumer guides, and established metrics, such as high-volume centers, as surrogates for high performance may not be suitable for identifying the preferred centers for patients contemplating elective surgery. Average outcome–based measures are more reliable than online consumer rankings but still far from optimal, and they did not result in lower TCOC. Our results suggest that a personalized approach based on precision navigation that uses readily available data to characterize a patient’s medical complexity in the context of individual hospitals may be associated with substantial improvements in outcomes while also lowering TCOC. An additional policy concern is that any mechanism designed to encourage patients to go to specific hospitals should ideally balance hospital use and capacity. Prioritizing hospital choice based on the typical static rating approaches would lead to a greater concentration of surgeries in a small number of top hospitals, thus overwhelming certain hospitals while underutilizing others. The personalized approach distributed patients over more hospitals (54 hospitals) than any of the other approaches (27 or fewer hospitals). Finally, the low proportion of patients that were matched to top hospitals using the precision navigation–based approach (<30%) presents a substantial opportunity for improving outcomes and costs for many patients by steering them to hospitals that are best suited for them.

One of the limitations of this study is the incomplete characterization of each patient’s unique medical needs and medical history, since only CMS administrative claims were available. Ideally, the online ratings would be based on reviews collected before the date of the procedure. However, access to historic ratings for online web-based systems was not available. Furthermore, the use of ED visits and postprocedure hospitalizations as outcomes does not reflect on the quality of life improvement following elective surgery that patients ultimately seek. This study was retrospective in nature and thus only showed associations between outcomes and various ranking and rating methods rather than suggesting causal relationships between ranking methods and patient outcomes. The ranking methods that were evaluated in this study’s experimental design may not have been intended to be used as stand-alone referral services or offer advice on the suitability or choice of particular hospitals. Rather, they may have been intended to be used (where applicable) as additional information that can be factored in with other inputs (such as referral recommendations provided by patients’ care providers) in choosing a hospital site for a future surgery. This study only evaluated the association of these ranking methodologies within the experimental framework considered, and the findings here should not be taken as a statement of their suitability for any purpose. Patients’ choices in elective surgeries can be influenced by socioeconomic factors as well as factors beyond their control, such as the availability of surgeons, recommendations from their care providers, and the network limitations of their health insurance plans.

The present study was performed using Medicare beneficiary claims data that primarily comprised older patients, and their applicability to younger patients with private health care insurance or Medicaid requires further research. Another limitation of this work was that the analysis was restricted to the greater Chicago metropolitan area. Future work is needed to extend this study to other procedures, non-Medicare populations, and hospitals nationwide. A prospective trial is warranted to further study the impact of hospital-ranking approaches on patient outcomes and total cost of care.

## Abbreviations

CMS: Centers for Medicare & Medicaid Services
ED: emergency department
FFS: fee-for-service
TCOC: total cost of care
